# A culture-independent nucleic acid diagnostics method for use in the detection and quantification of *Burkholderia cepacia* complex contamination in aqueous finished pharmaceutical products

**DOI:** 10.1371/journal.pone.0303773

**Published:** 2024-05-16

**Authors:** Huong Duong, Elizabeth Minogue, Shannon Fullbrook, Thomas Barry, Kate Reddington

**Affiliations:** 1 Nucleic Acid Diagnostics Research Laboratory (NADRL), School of Biological and Chemical Sciences, University of Galway, Galway, Ireland; 2 Microbial Diagnostics Research Laboratory, School of Biological and Chemical Sciences, University of Galway, Galway, Ireland; ENEA Casaccia Research Centre: ENEA Centro Ricerche Casaccia, ITALY

## Abstract

The *Burkholderia cepacia* complex (Bcc) is the number one bacterial complex associated with contaminated Finished Pharmaceutical Products (FPPs). This has resulted in multiple healthcare related infection morbidity and mortality events in conjunction with significant FPP recalls globally. Current microbiological quality control of FPPs before release for distribution depends on lengthy, laborious, non-specific, traditional culture-dependent methods which lack sensitivity. Here, we present the development of a culture-independent Bcc Nucleic Acid Diagnostic (NAD) method for detecting Bcc contaminants associated with Over-The-Counter aqueous FPPs. The culture-independent Bcc NAD method was validated to be specific for detecting Bcc at different contamination levels from spiked aqueous FPPs. The accuracy in Bcc quantitative measurements was achieved by the high degree of Bcc recovery from aqueous FPPs. The low variation observed between several repeated Bcc quantitative measurements further demonstrated the precision of Bcc quantification in FPPs. The robustness of the culture-independent Bcc NAD method was determined when its accuracy and precision were not significantly affected during testing of numerous aqueous FPP types with different ingredient matrices, antimicrobial preservative components and routes of administration. The culture-independent Bcc NAD method showed an ability to detect Bcc in spiked aqueous FPPs at a concentration of 20 Bcc CFU/mL. The rapid (≤ 4 hours from sample in to result out), robust, culture-independent Bcc NAD method presented provides rigorous test specificity, accuracy, precision, and sensitivity. This method, validated with equivalence to ISO standard ISO/TS 12869:2019, can be a valuable diagnostic tool in supporting microbiological quality control procedures to aid the pharmaceutical industry in preventing Bcc contamination of aqueous FPPs for consumer safety.

## Introduction

Pharmaceutical manufacturers are required to ensure all Finished Pharmaceutical Products (FPPs) meet Good Manufacturing Practice (GMP) standards for quality, safety, and efficacy. FPPs must undergo microbiological quality control testing to guarantee that they are safe to be released to consumers [1]. At the time of distribution, some FPPs (i.e. non-sterile types) are allowed to have a minimal microbial population present. However, they must be completely free of objectionable microorganisms, including microorganisms that can cause either damage to the product itself or potentially harm the consumer, like *Burkholderia cepacia* complex (Bcc) species [2].

Despite the widespread use of microbiological quality control testing within the pharmaceutical industry, bacterial contamination of FPPs remains a major challenge. According to recent published scientific publications and surveys, members of the Bccare the most frequently isolated bacterial contaminants in both sterile and non-sterile FPPs worldwide, particularly in relation to non-sterile aqueous products [3, 4]. Bcc contamination has been reported in a diverse range of aqueous, non-sterile FPPs, including oral pharmaceuticals, eye drops, nasal sprays, mouthwashes, skin creams and deionized water [5–10]. Bcc contamination in FPPs can either be intrinsic (occurring during the product’s manufacturing) or extrinsic (introduced in association with product use, e.g., diluted with non-sterile water, or transferred to storage in non-sterile container). Intrinsic Bcc contamination is particularly concerning as it potentially facilitates the spread of FPPs associated Bcc infections beyond a single hospital or even between various regions worldwide [11].

The Bcc comprises 27 closely related and distinct bacterial species that are found widely in nature and artificial habitats [12]. They are human opportunistic pathogens that can cause severe infection in immunocompromised and critically ill individuals [13]. Furthermore, recent reports have signalled the emergence of Bcc and Severe Acute Respiratory Syndrome Coronavirus-2 co-infections, with a growing concern that Bcc co-infection may contribute to an increased degree of disease severity in these Covid-19 patients [14, 15]. Therefore, the inadvertent use of Bcc contaminated FPPs constitutes a public health hazard with potential serious health implications for susceptible individuals and vulnerable populations [4]. In fact, various Bcc contaminated FPPs have been reported as the cause of several serious healthcare associated infection outbreaks across multiple healthcare settings [6, 16]. Moreover, the consequences of Bcc contamination in FPPs can result in company associated financial loss and reputational damage [17]. Over the last ten years, we estimate that at least 50 pharmaceutical companies worldwide have been impacted by Bcc contamination FPP recall related events [18–20].

In the context of Bcc contamination of FPPs and their potential to infect patients, the Food and Drug Administration (FDA) propose the inclusion of these bacteria in the “Objectionable Microorganisms” category [21]. This includes microorganisms that can cause either damage to the product itself or potentially harm the consumer [22]. In 2017 and in 2021, the FDA issued an advisory note to pharmaceutical companies about Bcc contamination hazards and specifically those associated with aqueous, non-sterile pharmaceutical manufacturing processes and FFPs [23]. The FDA further advised pharmaceutical drug manufacturers to monitor product components, processes and FPPs for Bcc bacteria. However, Bcc continues to avoid microbiological quality control and detection systems currently used by the industry sector. The persistence of Bcc in FPPs can be attributed to failures in GMP, inadequate factory cleaning procedures, the use of unsuitable grade of water for manufacturing, and to poor water distribution design and control systems [24].

Moreover, Antimicrobial Preservatives (APs) routinely incorporated into FPPs to prevent the proliferation of microbial contaminants are largely ineffective against Bcc species [25, 26]. In fact, members of the Bcc have been reported to utilise pharmaceutical APs as energy sources, allowing them to remain viable within an otherwise unfavourable environment [21]. The ability of Bcc to persist in the presence of APs is evidenced by numerous FPP contamination events in which Bcc have been isolated from opened and unopened containers of antimicrobial solutions, preserved pharmaceuticals, preserved cosmetics, and toiletries, at relatively high Colony Forming Units (CFU) (up to 10^5^−10^6^ CFU/mL) [6, 15]. Additionally, undetected Bcc in FPPs, largely reflects upon the limitations of current conventional microbiological quality control testing methods used within the pharmaceutical sector. These methods do not provide adequate specificity and sensitivity to detect Bcc at the quality control stage before releasing the FPP to the marketplace [4, 24].

In microbiological testing laboratories, examination of FPPs before market release relies on standard culture-dependent methods that are compliant with the USP<61> and USP<1113> for the isolation and identification of bacterial contaminants [27, 28]. These methods rely upon the growth of microorganisms; however, the recovery of microorganisms by culture can be challenging and ambiguous test results have been reported [29, 30]. During pharmaceutical manufacturing processes, microorganisms are also exposed to long periods of unfavourable growth conditions, such as low nutrient environments. Consequently, in order to adapt to these environmental stresses, bacteria may enter a viable but non culturable state, making them undetectable using culture [31]. Furthermore, culture-dependent microbiological testing of FPPs are subject to additional complexity due to the presence of APs. The antimicrobial properties of APs often render bacterial growth-based detection ineffective. As such, an AP neutralizer is required to be added to the FPP to ensure reliable bacterial culture testing analysis [28, 32]. This adds further cost and complexity to culture-dependent testing regimes currently in use in the industrial sector. Additionally, culture-dependent methods are time consuming because of the extended incubation times required for growth-based detection, bacterial enumeration, and identification methods, resulting in substantial delays in FPP release [21]. A recent benchmarking survey of 15 US FDA registered, GMP quality control (QC) laboratories in the pharmaceutical industry reported the overall quality testing operation cycle ranged from 5–34 days, with the testing time ranging from 3–15 days [33].

In 2019, the US Pharmacopeia published a specific qualitative culture-dependent test to determine the presence of Bcc in non-sterile FPPs–USP<60> “Microbiological Examination of Non-sterile Products–Tests for *Burkholderia cepacia* Complex”, in which an enrichment culture step is initially required, followed by growth on selective media BCSA [34]. This method can be problematic as the recommended culture growth media is not 100% selective for Bcc as it allows the growth of non-Bcc microorganisms [35]. In addition, this method is subject to the inherent limitations of culture-dependent methods such as being lengthy, labour intensive, and having relative low sensitivity and lacking specificity [36].

To prevent the potential for serious healthcare associated Bcc infection outbreaks and subsequent negative impacts on the pharmaceutical industry, manufacturers need to consider implementing more rapid, robust, and validated technologies for greater microbiological quality control evaluation of FPPs. The use of culture-independent nucleic acid diagnostics (NAD) technologies have recently emerged as promising alternatives for the detection of Bcc contamination [37–39].

To date, there have been several peer-reviewed publications describing the development of Nucleic Acid Diagnostic (NAD) methods for detecting and identifying Bcc in FPPs [37–41]. However, these studies have limitations. Most of the described methods were qualitative rather than quantitative in their approach. Many required a pre-enrichment growth step prior to using molecular NAD testing methods (such as PCR, qPCR or *16S rRNA* gene sequencing) for specific Bcc identification. The use of a pre-enrichment step increases turnaround time to result, removes the ability to perform absolute quantification and also adds significant test costs [40–42]. Furthermore, previously described qPCR methods do not incorporate an internal amplification control (IAC). These tests do not meet the requirements of recommended guidelines such as Minimum Information for Publication of Quantitative qPCR Experiments (MIQE) therefore, could be subject to technical errors and false negative results compromising the accuracy of results generated by these published NAD methods [37, 43].

Recently, we have published a study focused on the design and development of a novel specific Bcc qPCR NAD assay for the rapid, quantitative detection of all Bcc species [44]. The developed Bcc qPCR NAD assay was designed to meet the recommended MIQE guidelines [43]. It was validated as a component of a culture-independent Bcc NAD method for testing water used in pharmaceutical manufacturing with equivalence to ISO standard ISO/TS 12869:2019 [45].

In this study, we have applied the published Bcc qPCR NAD assay for the development and verification of a culture-independent NAD method to detect the Bcc in aqueous FPPs [44]. Since pharmaceutical water is the most common source of Bcc contamination, we focused our study on testing aqueous FPPs, which represent a significant risk of being contaminated by Bcc during their manufacture [31]. The performance of the developed culture-independent Bcc NAD method was compared with that of the recommended conventional Bcc culture-dependent method that is widely used in pharmaceutical manufacturing microbiological testing and control laboratories.

## Methods

### Culture and preparation of *B*. *cepacia* spiking suspensions

The validated Bcc qPCR NAD assay (S1 Table) used in this study was previously demonstrated to consistently detect all members of the Bcc [44]. The *B*. *cepacia* type strain (DSM 7288) was used as a representative strain of Bcc throughout this study. This strain was purchased from the German Collection of Microorganisms and Cell Cultures (Deutsche Sammlung von Mikroorganismen und Zellkulturen GmbH, DSMZ. Braunschweig, Germany).

Single *B*. *cepacia* DSM 7288 colonies that were less than 72hr old were inoculated in R2A broth (Lab M, Neogen Company, UK) at 30°C and shaken at 200 rpm until the culture reached an optical density (OD_600nm_) of 1 (demonstrating log phase growth), which corresponds to approximately 3x10^8^ CFU per 1 mL broth culture as determined by plate count. *B*. *cepacia* cells were harvested from culture aliquots by centrifugation at 4000 rpm for 8 min, followed by washing twice in sterile PBS (Oxoid, Basingstoke, UK). *B*. *cepacia* cells were then resuspended in PBS and adjusted back to OD_600nm_ 1. Subsequently, a series of ten-fold dilutions from the OD_600nm_ 1 culture (ranging from 10^−1^ to 10^−7^) were prepared in PBS and used as spiking suspensions for the artificial contamination of either AP solutions or aqueous FPPs. The OD_600nm_ 1 culture represented the 10^0^ spiking suspension. The actual quantity of *B*. *cepacia* within each prepared spiking suspension was determined at the time of use, using the spread plate technique (CFU per 1 mL) and the Bcc qPCR NAD assay (Genome Equivalents (GE) per 1 mL).

### AP solutions and over the counter (OTC) aqueous FPPs used in this study

#### AP solutions used in this study

Four APs that are frequently used in a wide range of OTC aqueous FPPs: cetylpyridinium chloride (CC), methylparaben (MP), sodium benzoate (SB), phenoxyethanol (PE) (Fisher Scientific) were used in this study. These four APs are representatives from different classes of APs (i.e., quaternary ammonium compounds, parabens, acids and their salts, and alcohols, respectively).

CC, SB and PE were prepared in HPW (Sigma-Aldrich). As MP is a water insoluble agent, stock solution of 5% w/v MP was prepared in propylene glycol (heated to approximately 45°C to dissolve completely) and subsequently used to prepare additional concentrations in HPW where required. All aqueous AP solutions were prepared on the day of use, sterilized using sterile 0.2 μm cellulose acetate syringe filters (VWR International), to achieve their highest recommended usage concentrations: 0.5% w/v CC; 0.4% w/v MP; 0.5% w/v SB; 1% v/v PE.

### OTC aqueous FPPs evaluated in this study

*i) OTC aqueous FPP types assessed*

Various types of commercially available aqueous FPPs were sourced locally. Seven product types, which included two non-sterile mouthwashes, a non-sterile nasal spray, a sterile single-use vial eye drop, a sterile eye spray, a non-sterile oral food supplement and a non-sterile skin toner were used in this study (Table 1). These products represented a range of aqueous FPPs previously shown to be susceptible to contamination by Bcc and have led to many healthcare associated infections in recent years [5–10].

**Table 1:** OTC aqueous FPPs analysed in this study.

Aqueous FPP type	Route of administration [46]	Package type	Sterility status	AP(s) constituents
Eye drops	Ocular	Single-use vial	Sterile	None
Eye spray	Ophthalmic	Multi-dose vial	Sterile	One AP: phenoxyethanol
Mouthwash 1	Oromucosal	Multi-dose vial	Non-sterile	Three APs: methylparaben, propylparaben, cetylpyridinium chloride
Mouthwash 2	Oromucosal	Multi-dose vial	Non-sterile	Two APs: sodium benzoate, cetylpyridinium chloride
Nasal spray	Nasal	Multi-dose vial	Non-sterile	None
Oral food supplement	Oral	Multi-dose vial	Non-sterile	One AP: potassium sorbate
Skin toner	Cutaneous	Multi-dose vial	Non-sterile	Three APs: methylparaben, sodium benzoate, phenoxyethanol

*ii) OTC aqueous FPPs preparation prior to culture-dependent evaluation*

For the culture-dependent Bcc analysis, aqueous FPPs were pre-treated with a solution of 4% Tween 20 (Merck) to neutralize the antimicrobial activity of APs incorporated during the manufacturing process as recommended by the compendia [28, 32, 38, 40]. Briefly, a dilution 1:10 of each aqueous FPPs was prepared by aseptically adding 1 mL of each product type to 9 mL of sterile USP buffered peptone water (Oxoid, Basingstoke, Hampshire, UK) containing 4% Tween 20.

*iii) Generating contaminated OTC aqueous FPPs with B*. *cepacia for use with culture-dependent and culture-independent NAD analysis*

Artificially contaminated aqueous FPPs were prepared by inoculating with *B*. *cepacia* spiking suspensions (as described in Section “Culture and preparation of *B*. *cepacia* spiking suspensions”). Depending on the aim of the analysis, different spiking suspensions were used for the product spiking procedure to obtain the desired *B*. *cepacia* concentration (GE per 1 mL of original product). The volume of the spiking suspension was 1% of the sample volume: 0.1 mL aliquots of each spiking suspension were inoculated into 10 mL of each prepared 1:10 aqueous FPP (the equivalent of 1 mL original aqueous FPP). For statistical reliability, each spiking experiment was performed in triplicate for each aqueous FPP. A non-spiked sample was included as a negative control for each aqueous FPP.

### Evaluation of bacterial contamination associated with the OTC aqueous FPPs

The intrinsic bacterial contamination population in aqueous FPPs (sealed and previously unopened products) was assessed via membrane filtration, bacterial culturing, bacterial isolation and bacterial identification by *16S rRNA* gene sequencing [27].

Each AP neutralized aqueous FPPs (10 mL) corresponding to 1 mL of original product was subjected to membrane filtration technique and culture according to the procedure as described in the USP<61> “Microbiological examination of nonsterile products” [28]: 10 mL of each spiked aqueous FPP was filtered immediately through a 0.4 μm hydrophilic polycarbonate membrane of 47 mm diameter (Isopore membrane filters, Merck Millipore) using a vacuum pump. The membrane filter was subsequently rinsed with three aliquots of 100 mL of sterile 0.1% peptone water to cleanse it of residual solution and adhering APs. After rinsing, each membrane filter was aseptically transferred, with the contaminated side facing upwards, onto the non-selective Tryptic Soy Agar (TSA) plate for the recovery of bacteria. The plates were incubated at 30°C for 3–5 days.

After incubation, visible colonies were isolated and cultured separately. Genomic DNA (gDNA) from each bacterial isolate was extracted using the Quick-gDNA MiniPrep Kit (Zymo Research). Conventional PCR for amplification of a segment of the *16S rRNA* gene using the primers 530F and 907R was performed using gDNA extracts [47]. PCR amplicons were purified using the High Pure PCR product purification kit (Roche Diagnostics) and sequenced externally (Sequiserve, Vaterstetten, Germany). The identity of bacterial isolates from aqueous FPPs was determined by analysing *16S rRNA* gene sequences compared to sequences available in GenBank using nucleotide BLAST analysis (https://blast.ncbi.nlm.nih.gov/Blast.cgi).

### Determination of CFU enumeration of *B*. *cepacia* spiked OTC aqueous FPPs via conventional culture

Spiked aqueous FPPs were prepared (as described in Section “OTC aqueous FPPs evaluated in this study”) for use with conventional culture. Three spiking suspensions (10^−5^, 10^−6^ and 10^−7^) were used to artificially contaminate aqueous FPPs to obtain final *B*. *cepacia* concentrations in a range of 10^2^−10^3^ CFU, 10−10^2^ CFU, <10 CFU, respectively, per 1 mL product. These *B*. *cepacia* concentration levels were chosen as the expected *B*. *cepacia* quantities are near or within the culture countable range.

*B*. *cepacia* spiked aqueous FPPs were subjected to membrane filtration technique and culture using the same procedure for non-spiked aqueous FPPs (as described in Section “Evaluation of bacterial contamination associated with the OTC aqueous FPPs”). After incubation, visible colonies were enumerated and reported. Those plates which exhibited colonies within the countable range of 20–80 CFU/membrane [29] were expressed as CFU per 1 mL of original product. Those that exceeded the range were referred to as “too numerous to count” (TNTC). If colonies were present but below the recommended countable range, they were counted and reported as an estimated count.

### The Bcc qPCR NAD assay for the specific quantitative detection of Bcc gDNA

#### The validated Bcc qPCR NAD assay

The Bcc qPCR NAD assay used in this study previously described (dx.doi.org/10.17504/protocols.io.4r3l223wql1y/v2) [44]. The Bcc qPCR NAD assay allows for the specific detection and absolute quantification of all 24 Bcc species. Three additional *Burkholderia* species (*B*. *orbicola*, *B*. *sola*, *B*. *semiarida*) have recently been added to the Bcc. As these Bcc members were not previously empirically demonstrated to be detected by the Bcc assay developed, *in silico* and BLAST analyses were performed to examine the possibility for these Bcc species to be detected using the diagnostics target *smpB* gene identified in the published study [44]. Nucleotide sequence within the *smpB* gene for these three Bcc species was retrieved from the National Center for Biotechnology Information (NCBI) website (http://www.ncbi.nlm.nih.gov). BLAST analysis (https://blast.ncbi.nlm.nih.gov/Blast.cgi?PAGE_TYPE=BlastSearch&BLAST_SPEC=MicrobialGenomes) comparing these nucleotide sequences and the nucleotide sequences of qPCR oligonucleotide primers and probes demonstrated matching 100% sequence identity. As such the three added Bcc members should also be detected using the published Bcc qPCR NAD assay. BLAST analysis of the Bcc specific probes also confirmed no potential cross-reactivity with non-Bcc microbial species’ nucleotide sequences available in the NCBI database.

The Bcc qPCR NAD assay was carried out as previously described [44]. Absolute Bcc GE quantities are extrapolated by comparison to the Bcc qPCR NAD assay calibration curve, using the threshold cycle (Ct) values for each qPCR reaction. The Limit Of Detection (LOD) and Limit Of Quantification (LOQ) were previously verified at 3 GE per qPCR reaction (with ≥ 90% probability) and 20 GE per qPCR reaction, respectively. To eliminate false negative results, an IAC targeting a synthetic construct sequence was incorporated into the Bcc qPCR NAD assay [44].

#### Evaluation of the performance of the Bcc qPCR NAD assay in the presence of Aps

To examine the impact of APs on the performance of the Bcc qPCR NAD assay, artificially contaminated AP solutions spiked with known Bcc concentrations were analysed by the Bcc qPCR NAD assay.

Two *B*. *cepacia* concentrations were chosen to spike the AP solutions, representing concentrations close to the median quantifiable range (10^3^−10^4^ Bcc GE/qPCR reaction) and close to the LOD (3 Bcc GE/qPCR reaction) of the Bcc qPCR NAD assay. As such, 10 μL aliquots of spiking suspensions 10^−1^ and 10^−4^ were used to spike 1 mL of each AP solution. Spiking suspensions were also used to inoculate 1 mL of PBS to be used as reference samples. Each spiking experiment was performed in triplicate for statistical reliability. Subsequently, bacterial gDNA from 0.1 mL of the spiked PBS reference and AP solutions was extracted and purified using the Quick-gDNA MiniPrep Kit (Zymo Research), as per the manufacturer’s instructions. The DNA was eluted in a final volume of 25 μL nuclease-free water. The eluted DNA extracts were directly used as templates in the Bcc qPCR NAD assay. The absolute quantity of Bcc GE detected in each qPCR reaction was determined by comparison to the established Bcc qPCR NAD assay calibration curve (i.e. y = -3.224x + 39.46, where *x* is the log_10_ of the starting quantity and *y* is the Ct value) [44].

The effect of APs on the detection and quantification of Bcc by the Bcc qPCR NAD assay was determined by comparing the qPCR-derived Ct value obtained for each spiked AP solution against that of the corresponding spiked PBS reference sample. Statistical analyses were performed using two-tailed *t*-test; the variations were considered statistically significant when *p* value < 0.05 [48]. All statistical calculations were performed using the Data Analysis ToolPak in Microsoft Excel software (Microsoft Inc., USA).

### Development of a culture-independent Bcc NAD method for use with OTC aqueous FPPs

For testing of aqueous FPPs, some minor modifications have been introduced to the culture-independent Bcc NAD method for the detection and quantification of Bcc from water (dx.doi.org/10.17504/protocols.io.rm7vzxox8gx1/v2) [44]. Briefly, the culture-independent Bcc NAD method for testing aqueous FPPs consists of 4 steps: 1) aqueous FPP preparation; 2) membrane filtration of the aqueous FPP; 3) bacterial gDNA extraction and purification; 4) the Bcc detection and quantification utilizing the Bcc qPCR NAD assay.

Note, since results for the microbiological examination of FPPs are routinely reported for 1 g or 1 mL of the product, the amount of aqueous FPPs to be used experimentally was set at 1 mL [49].

Step 1: *Aqueous FPP preparation*:

Each aqueous FPP was diluted 1:10 using sterile PBS to reduce the viscosity and increase the final sample volume to 10 mL such that membrane filtration could be performed.

Step 2: *Membrane filtration*:

Each diluted aqueous FPP (10 mL), corresponding to 1 mL of original product, was concentrated by filtration through a 0.4 μm hydrophilic polycarbonate membrane of 47 mm diameter (Isopore membrane filters, Merck Millipore), a filter with a low absorption capacity for extracellular nucleic acids [50], using a vacuum pump. The filtration cup was rinsed with 20 mL PBS to avoid adherence of bacterial cells to the filtration vessel walls. Each filter was then aseptically placed into a sterile 20 mL tube.

Step 3: *Bacterial gDNA extraction from the filter*:

Genomic Lysis Buffer (Quick-gDNA MiniPrep Kit, Zymo Research) (2 mL) was added to each filter and incubated at room temperature on a rolling platform for 30 mins. The remainder of extraction protocol was performed as per the Quick-gDNA MiniPrep Kit (Zymo Research) manufacturer’s instructions. The DNA was eluted in a final volume of 25 μL nuclease-free water.

Step 4: *The detection and quantification of Bcc by the validated Bcc qPCR NAD assay*:

The eluted DNA extracts were used as template in the Bcc qPCR NAD assay, as described in Section “The validated Bcc qPCR NAD assay”. Each DNA extract was tested in triplicate using the Bcc qPCR NAD assay. The absolute quantity of Bcc GE within each tested aqueous FPP was calculated based on the extrapolated Bcc GE quantities between qPCR technical replicates, the conversion factor (i.e., 5) between the volume of Bcc gDNA per qPCR reaction (5 μL) to the volume of total elution of Bcc DNA extracted (25 μL), and the initial tested aqueous FPP volume (1 mL). The results were expressed in Bcc GE per 1 mL of product.

The theoretical LOD and LOQ of the culture-independent Bcc NAD method (LODmeth, LOQmeth) for the detection and quantification of Bcc in aqueous FPPs were calculated based on the LOD and LOQ of the Bcc qPCR NAD assay, the initial tested aqueous FPP volume (1 mL) and the conversion factor between the quantity of Bcc GE per qPCR reaction to the quantity of Bcc GE per 1 mL original tested product. The theoretical LODmeth and LOQmeth were estimated to be 15 GE and 10^2^ GE per 1 mL product, respectively.

### Validation of the culture-independent Bcc NAD method for use with OTC aqueous FPPs

#### Validation strategy

To evaluate the overall accuracy and applicability of the culture-independent Bcc NAD method for the detection and quantification of Bcc contamination in aqueous FPPs, the method validation was performed by analysing artificially contaminated aqueous FPPs. *B*. *cepacia* spiked aqueous FPPs were prepared as described in Section “OTC aqueous FPPs evaluated in this study”. and subsequently analysed via the culture-independent Bcc NAD method (as described in Section “Development of a culture-independent Bcc NAD method for use with OTC aqueous FPPs”). HPW was used as a reference sample.

The performance characteristics of the culture-independent Bcc NAD method were evaluated considering the following parameters: accuracy, precision, robustness, and analytical sensitivity.

#### Validation of the culture-independent Bcc NAD method for the specific detection and quantification of Bcc in spiked OTC aqueous FPPs

The specificity of the developed culture-independent Bcc NAD method refers to the ability to specifically detect the target Bcc species of interest. This characteristic is conferred by the Bcc qPCR NAD assay component. The 100% specificity of the Bcc qPCR NAD assay was verified previously using Bcc gDNA and non-Bcc gDNA [44]. The performance of the culture-independent Bcc NAD method for the detection and quantification of Bcc in aqueous FPPs was further evaluated in this study in terms of accuracy, precision, robustness and analytical sensitivity using a range of reference HPW sample and aqueous FPPs artificially Bcc contaminated.

*i*. *Accuracy and precision of the culture-independent Bcc NAD method*
  The accuracy and precision of the culture-independent Bcc NAD method are two important parameters that describe the quality of quantitative data that can be generated for Bcc contamination in aqueous FPPs. Accuracy is the degree of closeness/agreement of a measured value generated by a specific procedure to the assumed actual or true value/reference; while precision is the degree of agreement between/among repeated measurements obtained by the same method under unchanged conditions [51].
  Firstly, the accuracy of the culture-independent Bcc NAD method was evaluated through a recovery experiment using HPW spiked with *B*. *cepacia* cells, comparing Bcc GE concentrations quantified and the actual values present in spiked HPW samples. The following spiking suspensions: 10^0^, 10^−4^, 10^−5^ were used in inoculating HPW samples to obtain final expected *B*. *cepacia* concentrations in a range of 10^7^−10^8^, 10^3^−10^4^, 10^2^−10^3^ GE per 1 mL of original product, respectively. These *B*. *cepacia* concentrations were chosen to cover the theoretical linear quantifiable range of the culture-independent Bcc NAD method, representing the Bcc GE concentration that the culture-independent Bcc NAD method would hypothetically detect and quantify.
  Due to the aqueous nature of the tested FPPs and a lack of defined acceptable recovery criteria for culture-independent Bcc NAD methods, the criteria for the recovery applied in our previous study testing Bcc in water sample was used [44]. This was in compliance with an ISO/TS 12869:2019 which is a widely use standard for the culture-independent NAD method for detecting *Legionella* species in water [45]. As such, the recovery of *B*. *cepacia* gDNA was calculated by the log10 difference between the *B*. *cepacia* GE quantity measured within spiked HPW samples after processing using the culture-independent Bcc NAD method and the initial spiked *B*. *cepacia* GE quantity. Recovery should have a value between -0.6log10unit to +0.3log10unit.
  Secondly, the precision of the culture-independent Bcc NAD method was evaluated by comparing the quantitative values measured, from each spiked Bcc GE concentration obtained between different experiments, conducted on different days. Precision of the quantitative measurements of the culture-independent Bcc NAD method, expressed in terms of percent Relative Standard Deviation (% RSD), was calculated based on log10-transformed *B*. *cepacia* GE concentration of repeated measurements at each *B*. *cepacia* spiked level on three different days. The acceptance criterion for precision is that the % RSD value should not exceed 25% [52].
*ii*. *Robustness of the culture-independent Bcc NAD method*
  Robustness of the culture-independent Bcc NAD method refers to the ability to maintain its performance when tested on different aqueous FPPs. Various types of aqueous FPP, as well as different product matrices, must not affect the culture-independent Bcc NAD method in terms of accuracy and precision. The same evaluation protocol applied to HPW was used for various *B*. *cepacia* spiked aqueous FPPs. The same criteria for acceptable accuracy, and precision, applied for HPW were used for each aqueous FPP at all spiking *B*. *cepacia* GE concentrations.
*iii*. *Analytical sensitivity of the culture-independent Bcc NAD method*
  To evaluate the analytical sensitivity of the culture-independent Bcc NAD method, the lowest detectable Bcc cells concentration in aqueous FPPs was determined. To this end, aqueous FPPs were inoculated with the spiking suspension 10^−6^ to obtain final expected *B*. *cepacia* concentration in a range of 10−10^2^ CFU per 1 mL of original product. This *B*. *cepacia* concentration was chosen as it was close to the theoretical LOD method of the culture-independent Bcc NAD method (15 Bcc GE per 1 mL product). To be accepted, ≥ 90% of samples must be detected.

## Results

### Evaluation of bacterial contamination associated with commercial OTC aqueous FPPs

The background bacterial contamination in aqueous FPPs prior to spiking with *B*. *cepacia* was assessed via membrane filtration followed by bacterial culturing, culture isolation and *16S rRNA* gene sequencing identification. The bacterial culture enumeration test of sterile aqueous FPPs detected no viable bacterial colonies (S2 Table). However, all non-sterile aqueous FPPs demonstrated the presence of between one to four Gram-positive bacterial species in each product albeit at relatively low levels (< 10 CFU/mL product) (S2 Table). This does not exceed the 100 CFU/mL limit of microbiological contamination for non-sterile aqueous FPPs recommended by USP [49].

There were no Bcc species detected for all previously unopened sterile and non-sterile aqueous FPPs by bacterial culture enumeration test and this was further confirmed by the culture-independent Bcc NAD method established in this study.

### CFU enumeration of *B*. *cepacia* spiked OTC aqueous FPPs via conventional culture

After confirming to be initially free from Bcc (as described Section “Evaluation of bacterial contamination associated with commercial OTC aqueous FPPs”), each aqueous FPP was artificially contaminated with spiking suspensions (10^−5^, 10^−6^ and 10^−7^) in triplicate, such that the final *B*. *cepacia* concentrations ranged from 6x10^0^ to 4.4x10^2^ CFU/mL. Spiked HPW was used as a reference sample. Total viable counts of each spiked HPW and aqueous FPP was assessed via membrane filtration, followed by bacterial culture enumeration testing for all *B*. *cepacia* spiking concentrations.

Results for the bacterial enumeration of all spiked aqueous FPPs are outlined in Table 2. HPW and aqueous FPPs spiked with *B*. *cepacia* spiking suspensions 10^−6^ and 10^−7^ demonstrated an average total viable count within a range of 13–25 and 2–10 CFU per 1 mL, respectively. All HPW and aqueous FPPs spiked with *B*. *cepacia* spiking suspension 10^−5^ fell outside of the countable range of the membrane filtration technique and were classified as TNTC.

**Table 2:** Bacterial enumeration of spiked OTC aqueous FPPs via conventional culture.

*B*. *cepacia* spiking suspension ^a^	Aqueous FPP	Spiked *B*. *cepacia* concentration ^b^ (CFU/mL)	Bacterial enumeration ^c^ (CFU/mL)
**10** ^**−5**^	HPW	**4.4x10** ^**2**^	TNTC
	Eye drops		TNTC
	Eye spray		TNTC
	Mouthwash 1		TNTC
	Mouthwash 2		TNTC
	Nasal spray		TNTC
	Oral food supplement		TNTC
	Skin toner		TNTC
**10** ^**−6**^	HPW	**2.7x10** ^**1**^	19±6 ^**d**^
	Eye drops		19±6 ^**d**^
	Eye spray		18±5 ^**d**^
	Mouthwash 1		14±1 ^**d**^
	Mouthwash 2		16±8 ^**d**^
	Nasal spray		15±2 ^**d**^
	Oral food supplement		16±3 ^**d**^
	Skin toner		17±1 ^**d**^
**10** ^**−7**^	HPW	**6x10** ^**0**^	5±2 ^**d**^
	Eye drops		6±3 ^**d**^
	Eye spray		5±2 ^**d**^
	Mouthwash 1		7±4 ^**d**^
	Mouthwash 2		6±3 ^**d**^
	Nasal spray		6±3 ^**d**^
	Oral food supplement		5±2 ^**d**^
	Skin toner		6±3 ^**d**^

^**a**^ *B*. *cepacia* spiking suspensions, from a series of ten-fold dilutions of the *B*. *cepacia* culture at OD_600nm_ = 1, used to spike into tested aqueous FPPs

^**b**^ Quantity of *B*. *cepacia* CFU in aliquots of spiking suspensions (confirmed by spread plate technique) used to spike the aqueous FPPs

^**c**^ Mean value ± standard deviation of the mean

^**d**^ estimated count, when bacterial enumeration exhibited colony counts below the countable range of the membrane filtration technique

TNTC: too numerous to count, when bacterial enumeration exhibited colony counts exceeded the countable range of the membrane filtration technique

### Evaluation of the performance of the Bcc qPCR NAD assay in the presence of APs

The evaluation of the possible impact of APs on the performance of the Bcc qPCR NAD assay was carried out by comparing the Bcc qPCR NAD assay results obtained for AP solutions against those of the corresponding PBS reference sample (without APs).

All PBS and AP solutions were inoculated with two spiking suspensions (10^−1^ and 10^−4^) such that a final *B*. *cepacia* concentration was estimated to be 3x10^3^ and 3x10^0^ GE per qPCR reaction, respectively. When inoculated with the *B*. *cepacia* spiking suspension 10^−1^, the measured Bcc GE concentrations for either PBS or AP solutions consistently remained within the same order of magnitude as the given spiked *B*. *cepacia* concentration. When inoculated with *B*. *cepacia* spiking suspension 10^−4^ containing a *B*. *cepacia* GE concentration lower than the LOQ of the Bcc qPCR NAD assay (15 GE per qPCR reaction), Bcc was detected in all PBS and AP solutions but could not be quantified accurately (Table 3).

**Table 3:** Detection and quantification of Bcc gDNA in the presence of APs by the Bcc qPCR NAD assay.

*B*. *cepacia* spiking suspension^a^	Theoretical Bcc GE/qPCR reaction	*B*. *cepacia* spiked aqueous FPP	Ct value^b^	Measured Bcc GE/qPCR reaction^c^	*P*-value on Ct values^d^
**10** ^**−1**^	3x10^3^	Reference sample-PBS	27.96 ± 0.09	3.7 x10^3^	**-**
		0.5% CC	27.93 ± 0.07	3.8 x10^3^	**0.57**
		0.4% MP	27.85 ± 0.2	4 x10^3^	**0.33**
		0.5% SB	27.87 ± 0.09	3.9 x10^3^	**0.13**
		1% PE	27.92 ± 0.28	3.8 x10^3^	**0.73**
**10** ^**−4**^	3x10^0^ (≥LOD, <LOQ)	Reference sample-PBS	38.19 ± 0.76	DBNQ	**-**
		0.5% CC	38.13 ± 0.49	DBNQ	**0.84**
		0.4% MP	38.54 ± 0.59	DBNQ	**0.12**
		0.5% SB	38.03 ± 0.75	DBNQ	**0.47**
		1% PE	38.00 ± 0.73	DBNQ	**0.5**

^**a**^ *B*. *cepacia* spiking suspensions, from a series of ten-fold dilutions of the *B*. *cepacia* culture at OD_600nm_ = 1, used to spike into tested samples

^**b**^ Mean value ± standard deviation of the mean

^**c**^ The absolute quantification from the obtained mean Ct values by comparison to the established calibration function of the Bcc qPCR NAD assay

^**d**^ Calculated using a two tail *t*-test comparing between Ct values obtained for each spiked AP solution against those of the corresponding PBS reference sample

DBNQ: detected but not quantified as the *B*. *cepacia* GE per qPCR reaction was lower than the LOQ of the Bcc qPCR NAD assay

The performance of the Bcc qPCR NAD assay for the detection and quantification of Bcc was evaluated by comparing the qPCR-derived Ct values for spiked AP solutions to its corresponding spiked PBS reference sample (Table 3). For PBS reference samples inoculated with *B*. *cepacia* spiking suspensions 10^−1^ and 10^−4^, Bcc DNA was detected by the Bcc qPCR NAD assay at Ct values 27.96 ± 0.09 and 38.19 ± 0.76, respectively. AP solutions inoculated with the *B*. *cepacia* spiking suspensions 10^−1^ and 10^−4^ demonstrated Ct values ranging from 27.85 ± 0.2 to 27.93 ± 0.07 and from 38.00 ± 0.73 to 38.54 ± 0.59, respectively. A two-tailed t-test analysis comparing Ct values obtained for each spiked AP solution and its corresponding PBS reference sample demonstrated that the observed Ct variations were not statistically significant (all p > 0.05) (Table 3). This demonstrates that no statistically significant difference in the Bcc qPCR NAD assay performance was observed when tested in the presence of APs.

### Detection and quantification of Bcc in spiked OTC aqueous FPPs via the culture-independent Bcc NAD method

*i*) *Accuracy and precision of the culture-independent Bcc NAD method*

The quantification limits of the culture-independent Bcc NAD method were evaluated in terms of accuracy and precision initially using spiked HPW reference controls. HPW was inoculated with three *B*. *cepacia* spiking suspensions (10^0^, 10^−4^, 10^−5^) to obtain Bcc concentrations that cover the theoretical quantifiable range of the culture-independent Bcc NAD method (4.9x10^7^ Bcc GE/mL; 4.6x10^3^ Bcc GE/mL; and 4.9x10^2^ Bcc GE/mL).

The accuracy in quantitative measurements of Bcc GE using the culture-independent Bcc NAD method was evaluated by recovery testing on spiked HPW. Specifically, the Bcc GE concentrations from spiked HPW samples were quantified using the culture-independent Bcc NAD method and subsequently compared to the actual spiked values. The difference between these two sets of values was calculated for each HPW sample spiked with three different *B*. *cepacia* concentrations and expressed in the log10 difference. The calculated recovery values ranged from -0.22 log10unit to -0.07 log10unit (Table 4). They all fell within the required range of -0.6log10unit to +0.3log10unit [45]. This demonstrated the closeness between the Bcc GE concentrations measured by the culture-independent Bcc NAD method and the actual Bcc GE concentrations initially introduced into the tested HPW samples.

**Table 4:** Recovery of Bcc detected from spiked OTC aqueous FPPs by the culture-independent Bcc NAD method.

*B*. *cepacia* spiking suspension^a^	Aqueous FPP	Spiked *B*. *cepacia* concentration^b^ (GE/mL)	Ct value^c^	Measured Bcc concentration^d^ (GE/mL)	%RSD	Log10 difference^e^
**10** ^**0**^	HPW	**4.9x10** ^**7**^	17.14 ± 0.13	4.2x10^7^	**0.52**	**-0.07**
	Eye drops		17.17 ± 0.24	4.1x10^7^	**0.99**	**-0.07**
	Eye spray		17.46 ± 0.24	3.4x10^7^	**0.98**	**-0.16**
	Mouthwash 1		16.73 ± 0.12	5.6x10^7^	**0.49**	**0.06**
	Mouthwash 2		16.95 ± 0.13	4.8x10^7^	**0.54**	**-0.01**
	Nasal spray		17.03 ± 0.15	4.6x10^7^	**0.59**	**-0.03**
	Oral food supplement		16.85 ± 0.15	5.2x10^7^	**0.61**	**0.02**
	Skin toner		16.69 ± 0.16	5.8x10^7^	**0.65**	**0.07**
**10** ^**−4**^	HPW	**4.6x10** ^**3**^	30.73 ± 0.69	2.8x10^3^	**6.2**	**-0.22**
	Eye drops		31.04 ± 1.48	2.8x10^3^	**13.3**	**-0.21**
	Eye spray		32.05 ± 1.04	1.2x10^3^	**10.5**	**-0.59**
	Mouthwash 1		32.02 ± 0.38	1.0x10^3^	**23.8**	**-0.52**
	Mouthwash 2		31.82 ± 0.55	1.2x10^3^	**5.5**	**-0.57**
	Nasal spray		31.04 ± 1.48	1.7x10^3^	**6.8**	**-0.43**
	Oral food supplement		30.35 ± 0.25	3.4x10^3^	**2.2**	**-0.13**
	Skin toner		30.79 ± 0.29	2.5x10^3^	**2.6**	**-0.27**
**10** ^**−5**^	HPW	**4.9x10** ^**2**^	33.77 ± 0.54	3.0x10^2^	**6.7**	**-0.21**
	Eye drops		33.88 ± 0.39	2.8x10^2^	**5.0**	**-0.25**
	Eye spray		34.99 ± 0.49	1.3x10^2^	**7.3**	**-0.59**
	Mouthwash 1		34.65 ± 0.96	1.8x10^2^	**13.6**	**-0.44**
	Mouthwash 2		34.63 ± 0.97	1.8x10^2^	**13.7**	**-0.43**
	Nasal spray		34.00 ± 0.35	2.7x10^2^	**9.2**	**-0.27**
	Oral food supplement		33.55 ± 0.08	3.4x10^2^	**0.9**	**-0.16**
	Skin toner		33.76 ± 0.52	3.1x10^2^	**6.5**	**-0.2**
**10** ^**−6**^	HPW	**2x10**^**1**^ **<LOQmethod (<10**^**2**^ **GE/mL)**	38.19 ± 0.19	DBNQ	**N/A**	**N/A**
	Eye drops		38.03 ± 1.41	DBNQ	**N/A**	**N/A**
	Eye spray		40.23 ± 1.61	DBNQ	**N/A**	**N/A**
	Mouthwash 1		38.86 ± 0.9	DBNQ	**N/A**	**N/A**
	Mouthwash 2		38.42 ± 1.11	DBNQ	**N/A**	**N/A**
	Nasal spray		38.54 ± 1.12	DBNQ	**N/A**	**N/A**
	Oral food supplement		38.26 ± 1.03	DBNQ	**N/A**	**N/A**
	Skin toner		38.92 ± 1.56	DBNQ	**N/A**	**N/A**

^**a**^ *B*. *cepacia* spiking suspensions, from a series of ten-fold dilutions of the *B*. *cepacia* culture at OD_600nm_ = 1, used to spike into tested samples

^**b**^ Quantity of *B*. *cepacia* GE in aliquots of spiking suspensions (measured by the Bcc qPCR NAD assay) used to spike aqueous FPPs

^**c**^ Mean value ± standard deviation of the mean

^**d**^ Quantity of Bcc GE detected in spiked aqueous FPPs

^**e**^ The difference between the quantity of spiked log10-transformed Bcc GE and the quantity of measured log10-transformed Bcc GE detected in spiked aqueous FPPs

%RSD: relative standard deviation, which is the percentage of the ratio of the standard deviation of the mean divided by the mean value of the log10-transformed Bcc GE measurements

DBNQ: detected but not quantified as the *B*. *cepacia* GE per qPCR reaction was lower than the LOQ of the Bcc qPCR NAD assay

N/A: not applicable. *Note*: %RSD and log10 difference were only calculated where spiked aqueous FPPs of each spiking level yielded quantifiable values

The precision of the Bcc quantitative measurements of the culture-independent Bcc NAD method was evaluated through the degree of agreement of measurements. This was expressed via the calculation of the %RSD, based on log10-transformed *B*. *cepacia* GE concentrations, between various repeated measurements on three separate days. %RSD was calculated for each HPW sample inoculated with three chosen *B*. *cepacia* spiking suspensions 10^0^, 10^−4^, 10^−5^ and was shown to be 0.52%, 6.2% and 6.7% respectively. They were all within the acceptance criteria of 25%, demonstrating the low variation between repeated measurements.

*ii*) *Robustness of the culture-independent Bcc NAD method*

To evaluate the robustness of the culture-independent Bcc NAD method for the quantitative detection of Bcc, various types of aqueous FPPs (with different ingredient matrices) artificially contaminated with *B*. *cepacia* were analysed. The results for the accuracy and precision of Bcc quantitative measurements of each spiked aqueous FPP are summarised in Table 4.

For each *B*. *cepacia* spiking suspensions (10^0^, 10^−4^, 10^−5^), the measured Bcc concentration for spiked aqueous FPPs consistently remained within the same order of magnitude as the given spiked *B*. *cepacia* concentration. Moderate variations between product types were observed but not exceeding one log step (i.e., <1.0 log10 range). The difference between the average Bcc GE concentration measured using the culture-independent Bcc NAD method and the actual value was calculated for each spiked aqueous FPP at all three *B*. *cepacia* spiking concentrations. The calculated recovery values expressed in the log10 difference, ranged from -0.59 log10unit to +0.07 log10unit, complying with the recovery criteria (Table 4).

To evaluate the precision of the culture-independent Bcc NAD method, the % RSD was calculated for each tested aqueous FPP at each spiking level. A high degree of precision was observed at a high spike concentration (i.e., 4.9x10^7^ *B*. *cepacia* GE per 1 mL product) when all % RSD < 1% for all tested aqueous FPPs. As the *B*. *cepacia* spiking concentration decreased, the %RSD increased; however, all %RSD values for the quantification of three *B*. *cepacia* concentrations in all tested product types complied with the acceptance criterion (≤ 25%) (Table 4).

The culture-independent Bcc NAD method detected Bcc DNA from all aqueous FPPs inoculated with *B*. *cepacia* at three tested spiking concentrations. All the non-spiked aqueous FPPs previously determined to be free from Bcc remained negative. This demonstrated that the culture-independent Bcc NAD method didn’t produce false positive results when *B*. *cepacia* was not present. The incorporated IAC was consistently detected in all Bcc qPCR NAD assay reactions for all aqueous FPPs, spiked and non-spiked, at Ct values ranging from 30.15–32.61, indicating that no qPCR reaction inhibition had occurred.

*iii*) *Analytical sensitivity of the culture-independent Bcc NAD method*

When all HPW and aqueous FPPs were inoculated with the spiking suspension 10^−6^ (prepared as outlined in methods section Culture and preparation of *B*. *cepacia* spiking suspensions), the final *B*. *cepacia* concentrations in each spiked sample were expected to be 2x10^1^ Bcc CFU/mL product. Bcc DNA was detected in all samples tested. This demonstrated the high analytical sensitivity of the culture-independent Bcc NAD method with detection of Bcc in all spiked aqueous FPPs as low as 2x10^1^ Bcc CFU/mL product. This is consistent with the theoretical LOD of the culture-independent Bcc NAD method (15 Bcc GE/mL product, ≥ 90% probability). Note, a lower Bcc concentration (< 20 Bcc CFU/mL) in aqueous FPPs could be detected by the culture-independent Bcc NAD method, however the probability of repeatable detection is lower than 90% [45].

## Discussion

Distributing FPPs contaminated with Bcc to customers represents a public health risk. Bcc contaminated aqueous FPPs are of great concern because they have been frequently reported as the source of severe patient infection and epidemics in healthcare settings over the past 20 years [21, 23]. Testing for the absence or presence of Bcc contamination in FPPs, especially aqueous FPPs, is an essential part of the microbiological risk assessment process prior to market release. The most common approach for the microbiological examination of FPPs relies on conventional culture techniques. However, the limitations of these culture-dependent diagnostics strategies are well documented. NAD technologies can overcome many of the limitations of current microbiological testing methods. This is due to relatively straightforward result interpretation, shorter turnaround time to result, higher sensitivity and more accuracy [53]. Reducing the turnaround time to result of microbiological analysis of FFPs is of paramount importance to the industry sector, as a more rapid turnaround time would shorten quarantine FPP periods, potentially ensuring an earlier release of the FPP to the market than is currently in place. Furthermore, earlier and more reliable detection of Bcc contamination events associated with aqueous FPPs could allow for the faster discovery of the contamination source during manufacturing, expediting the prompt intervention of contamination control strategies and the implementation of corrective measures required to eliminate the associated Bcc contamination [53].

The objective of this study was to adopt a qPCR NAD method incorporating a previously validated Bcc qPCR NAD assay for the specific and quantitative detection of Bcc in aqueous FPPs [44]. However, APs, which are frequently incorporated into commercial FPPs, have posed some challenges for several published qPCR NAD assays, as they can affect the integrity of gDNA isolated and also may cause an inhibitory effect on downstream qPCR NAD target amplification [54, 55]. Therefore, the potential impact on the performance of the Bcc qPCR NAD assay of four APs (i.e., CC, MP, SB and PE), which are representatives from different classes of APs frequently used within aqueous FPPs, was investigated. The quantitative detection capacity of the Bcc qPCR NAD assay was not impacted by any of the four tested AP solutions. Consequently, while culture-dependent methodologies often require the addition of an appropriate AP neutralizer to inactivate APs before a reliable microbiological growth-based examination, the Bcc qPCR NAD assay does not require this step, thereby further reducing the test complexity and potential cost [28, 32].

Aqueous FPPs, because of their nature, are amenable to membrane filtration microbial testing techniques, and this was employed with the developed culture-independent Bcc NAD method in this study. Polycarbonate membrane filters with low binding affinity for free extracellular DNA, compared to other typically used filter types, were used to avoid the overestimation of Bcc contamination caused by the detection of the presence of extracellular Bcc DNA from non-viable Bcc [50]. Although detecting Bcc DNA from non-viable, injured, or damaged Bcc intact cells cannot be completely ruled out using the developed culture-independent Bcc NAD method, the presence of these Bcc cells in tested aqueous FPPs could indicate a recent Bcc contamination event. If Bcc DNA in an FPP is detected using the developed method, appropriate analyses on the same samples can be performed to further investigate the viability of the microorganisms detected.

The Bcc qPCR NAD assay component of the culture-independent Bcc NAD method was previously demonstrated to be 100% specific for the detection of only Bcc species [44]. The high testing specificity observed is a significant advantage when compared to the recommended culture-dependent compendia tests, as the misidentification of Bcc with their closely related species by culture is frequently reported [56]. In fact, whether the non-selective media for bacterial enumeration of aqueous FFPs or specific selective media for Bcc is used, further tests are always required to identify suspected Bcc contamination after culture has been completed [27, 28, 34]. With the culture-independent Bcc NAD method presented, specific detection, identification and quantification results can be achieved in a single Bcc test in ≤ 4 hours.

From the perspective that Bcc must be completely absent from aqueous FFPs, qualitative interpretation results of quality control tests are sometimes more important than quantitative ones [57]. However, the relatively accurate quantitative detection using the developed culture-independent Bcc NAD method could provide manufacturers with important information on the severity of Bcc contamination and subsequently plan the appropriate remedial measures. The culture-independent Bcc NAD method presented in this study can detect and simultaneously quantify Bcc contamination levels from aqueous FPPs with accurate and precise measurements. A high degree of Bcc recovery was achieved from all aqueous FPPs spiked with different *B*. *cepacia* concentrations, indicating no significant loss of Bcc cells and purified Bcc DNA occurred during the entire method. Therefore, the Bcc GE concentrations quantified by the culture-independent Bcc NAD method are estimated to closely reflect actual CFU values present in the Bcc spiked aqueous FPPs. The agreement amongst several measurement values of the same Bcc concentrations with minimal variability further demonstrated the precision, reproducibility, and consistency in Bcc quantification of the culture-independent Bcc NAD method. This ability to provide accurate and precise results of Bcc contamination levels in aqueous FPPs, using the culture-independent Bcc NAD method, is superior to current culture-dependent methodologies, which are subject to inherent bias and limitations associated with culture [29].

Furthermore, the robustness of the culture-independent Bcc NAD method in terms of the quantitative detection of Bcc was examined on various types of spiked aqueous FPPs. OTC aqueous FPPs examined in this study have different routes of administration for end users (oral, nasal, ocular, transdermal) along with different ingredient matrices. Overall, the accuracy and precision results generated using the culture-independent Bcc NAD method for all spiked aqueous FPP types fall within the relevant acceptance criteria, indicating the aqueous FPP type did not significantly affect the Bcc quantitative detection capability. Note, OTC aqueous FPPs tested in this study contain either none, one, or more APs within each FPPs final formulation (Table 1). However, the presence of these APs within aqueous FPPs was further demonstrated not to have any significant impact on the performance of the culture-independent Bcc NAD method. Therefore, we consider that the culture-independent Bcc NAD method can be applied to many filterable aqueous FPP, regardless of the composition or the type of APs used by the manufacturers.

Another important limitation to consider with culture-dependent methods is the comparatively narrow quantifiable range (between 20 and 80 CFU/membrane) that can be achieved by culture enumeration. In this study, when membrane filtration and conventional culture techniques were used to enumerate bacterial cell numbers in aqueous FPPs spiked with three *B*. *cepacia* concentrations (10^−5^, 10^−6^, 10^−7^), the quantitative results for only one concentration (10^−6^) fell within the reliable culture countable range. Aqueous FPPs that exhibited plate counts either above or below the countable range were subjected to counting errors. When there were too many colonies present, the interpretation of Bcc CFU was affected as a result of confluence and overcrowding across the surface of the filter membrane. Where colony numbers were too low, minor errors in dilution technique could have a drastic effect on the final CFU result [29]. Hence, the CFU data for those samples outside of the countable range are inaccurate and unreliable. In fact, due to the relatively narrow culture countable range, the bacterial enumeration of an aqueous FFP often requires preparing a range of serial dilutions of the given aqueous FPP and subsequently performing multiple replicates for each dilution in an effort to obtain countable Bcc CFU. This is time consuming, requires more consumables, and contributes to delays in time to result [29]. In contrast, the developed culture-independent Bcc NAD method is quantitative over a range of six orders of magnitude (from 4.9x10^2^ to 4.9x10^7^ Bcc GE/mL). As such, this method allows for the direct measurement of Bcc GE numbers in aqueous FPPs with a minimum workload and experimental manipulation compared with Bcc culture-dependent techniques. The broad dynamic range of the developed culture-independent Bcc NAD method also suggests its potential application as a tool for the rapid response to Bcc contaminated aqueous FPP related outbreak incidents, which have been reported to result from Bcc contamination levels up to 10^5^−10^6^ CFU/mL [6, 15].

The use of low sensitivity, culture-dependent, quality control testing has been explained as one cause for the release of intrinsic Bcc contaminated aqueous FFPs [31, 58]. Many aqueous FPPs pass microbiological quality control testing for bioburden by not exceeding the 100 CFU/mL microbial limit and are released for distribution. However, they are later recalled due to Bcc contamination exceeding the acceptable bioburden level. This indicates initial evasion from culture-dependent detection with subsequent later growth and proliferation in the marketed product [31]. These cases further highlight the limitation of culture-dependent FPP release testing, which often fails to detect low levels of Bcc contamination. This is particularly worrisome due to the ability of Bcc, even when initially present at very low levels, to persist, grow and proliferate within preserved aqueous FFPs. This causes adverse effects on product properties and their potential performance in conjunction with the possibility for subsequent health hazards [21]. Considering the possible low level of Bcc contamination in aqueous FFPs at the time of release, in combination with their aptitude to evade culture-dependent detection, the sensitivity of the developed culture-independent Bcc NAD method was evaluated on aqueous FPPs spiked with low concentrations of Bcc (<100 CFU/mL). The culture-independent Bcc NAD method presented showed an ability to detect Bcc in all spiked aqueous FPPs at a considerably low concentration of 20 Bcc CFU/mL, which is consistent with the theoretical LOD method (15 Bcc GE/mL product, ≥ 90% probability) determined during the Bcc NAD method development and verification.

## Conclusion

Due to the limitations associated with Bcc culture-dependent methods outlined above, we consider the implementation of the culture-independent testing Bcc NAD method is of great relevance for delivering a robust microbiological quality control methodology for use with FPPs in the pharmaceutical industry sector [53]. The rapid culture-independent Bcc NAD method presented here, with all parameters in compliance with technical criteria for the validation of a NAD method, provides appropriate specificity, accuracy, precision, sensitivity, and faster turnaround time to result, ensuring the reliable detection and quantification of Bcc contaminants that maybe associated with OTC aqueous FPPs.

Moreover, this offers a valuable diagnostic tool, either complementary to or as an alternative to the recommended Bcc culture-dependent methodologies, in testing aqueous FPPs for Bcc contamination prior to product release and distribution to the market.

## Supporting information

S1 Table Oligonucleotide primers and probes used in the BCC qPCR assay. (DOCX)

S2 Table The background bacterial contamination enumeration of non-spiked OTC aqueous FPPs. (DOCX)
