# Supplementary material for: A culture-independent nucleic acid diagnostics method for use in the detection and quantification of Burkholderia cepacia complex contamination in aqueous finished pharmaceutical products
Source: PLoS One. 2024 May 16;19(5):e0303773. doi: 10.1371/journal.pone.0303773 (PMC11098509; doi:10.1371/journal.pone.0303773)
Supplement: S1 Table — (DOCX) [file pone.0303773.s001.docx]

**S1 Table. Oligonucleotide primers and probes used in the BCC qPCR assay**

| **Name** | **Gene target** | **Function** | **DNA sequence (5’-3’)** |
| --- | --- | --- | --- |
| Bcc F | *smpB* | Bcc forward primer | CRATCCHTTCATGAGCATCA |
| Bcc R | *smpB* | Bcc reverse primer | TTGACYTCCCAGCCYTC |
| Bcc P1 | *smpB* | Bcc specific hydrolysis probe -1 | 5HEX/ACAACAGGA/ZEN/ARGCGCACTTCG/3IABkFQ |
| Bcc P2 | *smpB* | Bcc specific hydrolysis probe -2 | 5HEX/CAACAGGAA/ZEN/AGCGCGCTTCG/3IABkFQ |
| SIAC F | Synthetic construct | IAC forward primer | ATGCCAGTCAGCATAAGGA |
| SIAC R | Synthetic construct | IAC reverse primer | CAGACCTCTGGTAGGATGTAC |
| SIAC P | Synthetic construct | IAC specific hydrolysis probe | 5Cy5/TCGGCACTA/TAO/CCGACACGAAC/3IAbRQSp |

/5HEX/ = 5' HEX (6-hexachlorofluorescein) fluorophore; /ZEN/ = ZEN™ internal quencher; /3IABkFQ/ = 3' Iowa Black® FQ terminal quencher; /5Cy5/ = 5' Cy5 (cyanine5) fluorophore; /TAO/ = TAO™ internal quencher; /3IAbRQSp/ = 3' Iowa Black® RQ-Sp terminal quencher
