# Supplementary material for: A culture-independent nucleic acid diagnostics method for use in the detection and quantification of Burkholderia cepacia complex contamination in aqueous finished pharmaceutical products
Source: PLoS One. 2024 May 16;19(5):e0303773. doi: 10.1371/journal.pone.0303773 (PMC11098509; doi:10.1371/journal.pone.0303773)
Supplement: S2 Table — (DOCX) [file pone.0303773.s002.docx]

**S2 Table. The background bacterial contamination enumeration of non-spiked OTC aqueous FPPs**

| **Aqueous FPP** | **CFU count ***  **(CFU/1 mL product)** | **Bacterial identification by *16S rDNA* sequencing** |
| --- | --- | --- |
| Eye drops | 0/0/0 | - |
| Eye spray | 0/0/0 | - |
| Mouthwash 1 | 0/8/1 | [*Kocuria sp.*](about:blank)  [*Micrococcus sp.*](about:blank) |
| Mouthwash 2 | 1/0/1 | [*Micrococcus sp.*](about:blank) |
| Nasal spray | 2/7/2 | [*Kocuria sp.*](about:blank)  [*Micrococcus sp.*](about:blank) |
| Oral food supplement | 0/3/1 | *Bacillus sp.*  [*Cytobacillus sp.*](about:blank)  [*Kocuria sp.*](about:blank)  [*Micrococcus sp.*](about:blank) |
| Skin toner | 0/2/1 | *Bacillus sp.*  *Naganishia sp.*  *Staphylococcus sp.* |

***** CFU(s) per plate from 3 replicates
